# Differences in comprehending and acting on pandemic health risk information: a qualitative study using mental models

**DOI:** 10.1186/s12889-022-13853-y

**Published:** 2022-07-29

**Authors:** Siv Hilde Berg, Marie Therese Shortt, Henriette Thune, Jo Røislien, Jane K. O’Hara, Daniel Adrian Lungu, Siri Wiig

**Affiliations:** 1 Centre for Resilience in Healthcare, Faculty of Health Sciences, University of Stavanger, Kjell Arholmsgate 43, 4021 Stavanger, Norway; 2 School of Healthcare, University of Leeds, Leeds, UK

**Keywords:** Mental models theory, Risk communication, COVID-19

## Abstract

**Background:**

A worldwide pandemic of a new and unknown virus is characterised by scientific uncertainty. However, despite this uncertainty, health authorities must still communicate complex health risk information to the public. The mental models approach to risk communication describes how people perceive and make decisions about complex risks, with the aim of identifying decision-relevant information that can be incorporated into risk communication interventions. This study explored how people use mental models to make sense of scientific information and apply it to their lives and behaviour in the context of COVID-19.

**Methods:**

This qualitative study enrolled 15 male and female participants of different ages, with different levels of education and occupational backgrounds and from different geographical regions of Norway. The participants were interviewed individually, and the interview data were subjected to thematic analysis. The interview data were compared to a expert model of COVID-19 health risk communication based on online information from the Norwegian Institute of Public Health. Materials in the interview data not represented by expert model codes were coded inductively. The participants’ perceptions of and behaviours related to health risk information were analysed across three themes: virus transmission, risk mitigation and consequences of COVID-19.

**Results:**

The results indicate that people placed different meanings on the medical and scientific words used by experts to explain the pandemic (e.g., virus transmission and the reproduction number). While some people wanted to understand *why* certain behaviour and activities were considered high risk, others preferred simple, clear messages explaining *what* to do and *how* to protect themselves. Similarly, information about health consequences produced panic in some interviewees and awareness in others.

**Conclusion:**

There is no one-size-fits-all approach to public health risk communication. Empowering people with decision-relevant information necessitates targeted and balanced risk communication.

**Supplementary Information:**

The online version contains supplementary material available at 10.1186/s12889-022-13853-y.

## Background

The COVID-19 pandemic has been characterised by scientific uncertainty regarding the aetiology and management of the disease [1, 2]. This uncertainty has posed a significant challenge for health authorities, who are required to convey information to the public even in the absence of scientific consensus. Under these difficult conditions, the understanding of mental models can be instructive [3]. Mental models are personal, inner images of external reality that people use to interact with the world [3, 4]. According to descriptive decision theory, mental models are used to explain how people make decisions based on how they perceive the surrounding world [3]. Mental models have been studied in relation to stakeholder knowledge about complex decision-making processes [5–7]. People’s mental models are drawn from their personal experiences, perceptions and understanding of the world, and this information is used to respond to risks [3].

There is an urgent need for more studies of pandemic health risk communication that consider the diversity of receivers [8]. The mental models approach to risk communication is one way of exploring the diversity of the receivers of communication, particularly their comprehension, interpretation and preferences [7]. The mental models approach might assist in the creation of effective and tailored risk communication by identifying what the audience already knows or believes about an issue and then supplying the decision-relevant information that their mental models require [7]. The mental model framework has been found to be useful in improving participants’ context-specific knowledge, scientific comprehension [9], attitudes [10] and self-reported behaviour [11]. It has also been applied to the study of nonexperts’ mental models of infectious health risks, such as sexually transmitted diseases and sexual risk behaviour [11, 12], vaccination [13–16] and influenza pandemics [17].

The classical normative decision theories emphasise how people should make their decisions, and the descriptive decision theories describe how people actually make their decisions. Mental models are a descriptive decision theory to gain insight into how people make decisions based on how they perceive their surrounding world [5]. However, the focus of mental model studies related to health risk has been largely on the content of communication (i.e., improving comprehension and knowledge and correcting misconceptions). However, to design effective risk communication interventions, the communication process also matters (i.e., preferred modes of communication, sources, and learning styles) [8, 18]. This study explored how people use mental models to make sense of scientific information and apply it to their lives and behaviours in the context of COVID-19. Specifically, our research question was “How does a sample of Norwegian citizens perceive, act on and learn about health risk related to COVID-19?”.

## Methods

The study approach was adapted from the mental models framework [7, 19]. The mental models approach by Morgan et al. [7] is a stepwise procedure starting with the creation of an expert model and a summary of the scientific literature and guidelines, followed by elicitation of decision makers’ (i.e., the public’s) mental models [19]. This study is part of a sequential mixed method effect study using mental models to study the effect of public video health communication [8, 20, 21]. To explore the public’s mental models in relation to the risk of COVID-19, we used semistructured interviews conducted between 3 February and 3 March 2021.

### The study context

Norway’s first case of COVID-19 was identified on 26 February 2020 [22]. Since the country’s first wave of COVID-19 in March–April 2020, the government’s strategy has been to limit the spread of infection using the Testing, Isolation, Infection Detection and Quarantine (TIDQ) strategy and occasionally closing schools and kindergartens and cancelling cultural events [23]. The government’s goal was to contain the spread of the virus so that the infection rate did not exceed the capacity of the health and care services and the municipal health service [24]. Among 48 European countries, at the time of data collection, Norway was 46^th^ in the number of total positive cases and 45^th^ in deaths per million from COVID-19 [25]. At the time of data collection (January-March 2021), no participants had received the vaccine, and the alpha variant of the coronavirus (B.1.1.7) had started to spread, leading to Norway’s third wave of COVID-19.

### Population and procedures

Instead of recruiting a large representative sample, it is recommended to recruit a small but diverse sample of participants (10–15 participants) when exploring mental models [19]. We purposively sampled participants for the individual interviews to cover a wide variety of mental models. People unable to provide informed consent (e.g., younger than 18 years of age and/or with severe health conditions) were excluded. The participants were recruited by invitation from nongovernmental organisations and using the research team’s extended network. All participants provided written and voluntary consent to participate. The study was approved by the Norwegian Centre for Research Data (Ref. nr 583,192).

We developed a semistructured interview guide with open-ended questions to explore the participants’ knowledge and comprehension of COVID-19 risk, perceived potential effects of COVID-19, self-protective measures, ways of learning about pandemic risk and issues of concern [7] (see Additional file 1).

The interview guide was tested in a pilot interview, and the data from this interview were included in the analysis. To comply with national COVID-19 measures on maintaining physical distance, the participants were interviewed using video conference software (Zoom) or by telephone. The interviews lasted a mean (range) of 50 (30–76) minutes. Occasional problems with sound were resolved by asking the participants to confirm the interviewer’s interpretations during the interviews.

### Interview guide development

The interview guide development was informed by previous mental models of infectious diseases spread by human-to-human transmission, including influenza [17, 26] and sexually transmitted infections/diseases [7, 11, 27]. These studies have covered topics about disease transmission, exposure, mitigation, and health effects, which were used as a framework for the interview guide. Furthermore, to design COVID-19 specific questions, we used information published on the web pages by the Norwegian Institute of Public Health (NIPH) Jan-March 2021 [23, 28–30]. We asked about their beliefs regarding virus transmission, exposure, consequences and health effects, risk comparison, contagiousness, exponential growth and the *R*-value. In addition, we asked the participants how they had learned about COVID-19. The interview guide is found in Additional file 1. The interview guide did not include the topic of vaccination since the interviews were conducted before people were vaccinated. Quarantine and isolation were excluded because their inclusion would have required extended ethical approval.

### Analysis

The interview data were analysed following de Bruins and Bostroms’ mental models methodological framework, which compares the lay model with the expert model [19]. When reconciling the expert and public mental models (lay model), we aimed to learn how experts and the public perceived information differently and to identify decision-relevant information that was missing in people’s mental models [19]. We were also interested in topics that the participants themselves identified.

The expert model was based on the NIPH webpages of Jan-March 2021, which consisted of up-to-date epidemiological knowledge published by the NIPH regarding COVID-19 and SARS-CoV-2 [30], as well as mitigation advice and measures [23, 28, 29].

SHB, SW, HT and MT read the transcripts and discussed their first impressions of the public interviews. SHB first deductively coded and organised the data material according to predefined codes derived from the expert model. Data not represented by the expert model were coded inductively [19]. Finally, SHB compared the expert model with the public model by displaying the codes derived from the public interviews and the key messages communicated by the NIPH in a table. SHB, SW, HT and MT discussed and validated the analysis.

## Results

The sample comprised 15 Norwegian citizens (*N* = 7 female and *N* = 8 male) who were between 19 and 79 years old, with 13–18 years of formal education, from different geographical regions of Norway and from diverse occupational areas. The sample also included students, people temporarily laid off, and retirees (Table 1).

**Table 1:** Sample Characteristics

No	Gender	Age	Education and occupation	City
1. (pilot)	F	32	Physiotherapist, temporarily laid off	Western city and the Netherlands
2	F	67	Nurse, retired	Eastern city
3	M	67	Engineer, retired	Small western city
4	M	49	Master’s degree, computer engineer	Western city
5	F	79	Nurse, manager, retired	Western city
6	F	65	Nurse assistant, retired	Eastern town
7	F	19	First-year university student	Northern city
8	F	19	First-year university student	Northern city
9	F	19	First-year university student and kindergarten assistant	Northern city
10	M	27	High school education, NGO worker	Northern city
11	M	37	High school education, firefighter	Small southern city
12	M	32	Airline pilot, temporarily laid off	Small southern city
13	M	51	Farmer, agronomist	Western town
14	M	34	Chef, offshore worker	Small eastern city
15	M	22	Electrician	Western town

Three themes emerged pertaining to the way in which the participants perceived and acted on health risk information related to COVID-19 (lay mental model): virus transmission, exposure to risk and consequences of COVID-19 (Table 2).

**Table 2:** Themes and Subthemes

Virus transmission	Risk mitigation	Consequences of COVID-19
Comprehending modes of virus transmission	Affecting exposure to risk	Building situational awareness
Understanding terms differently	Learning about mitigation in different ways	Perceiving personal health consequences differently
Acting on uncertain evidence	Being driven by symbolic values	Emphasising secondary consequences

### Virus transmission

The first theme, *virus transmission,* describes public perceptions related to how the virus enters the human body*.* It comprises three subthemes: comprehending modes of virus transmission, understanding terms differently, and acting on uncertain evidence*.*

#### Comprehending modes of virus transmission

The participants trusted the government’s advice on virus transmission because the national measures were based on expert advice. However, some expressed confusion over the government’s contradictory information about the health risk:

You do not have to have contact through body fluids to get it, so you do not have to drink from the same water bottle to get it. You can get it through breathing the same air. If you are infected, you exhale corona particles, and then I can breathe in and be infected that way. But now I am very insecure. This is where I have actually gotten mixed messages (No. 9, Female, 19 years old).

**Fig. 1:**
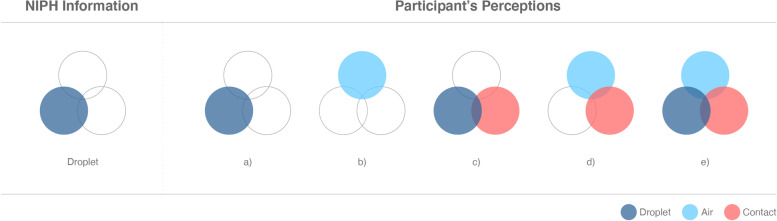
NIPH information about the main modes of COVID-19 virus transmission versus participants’ perceptions of virus transmission

A rich variety of perceived modes of transmission was described, including droplets, air and/or contact transmission. Some of the participants identified transmission modes not regarded as significant by the NIPH, such as food, clothes and faeces. NIPH singled out droplet transmission as the most important mode of transmission and communicated that airborne transmission and contact transmission existed but were nonsignificant means of transmission. However, many of the participants believed in multiple equally important means of transmission or identified air as the main route of transmission. The participants described transmission in ways that could be categorised as: a) mainly droplets; b) mainly through air (aerosol); c) a combination of droplets and contact; d) a combination of airborne and contact; or e) a combination of droplets, air and contact (Fig. 1).

Some of the participants showed an understanding of presymptomatic and asymptomatic transmission and emphasised the importance of this knowledge for their understanding of *why* they had to maintain distancing even from family members and *why* people had to quarantine. However, the participants did not necessarily receive this information from the government but rather from friends.

Some missed this information regarding how the virus entered the human body to understand how they could better protect themselves:

How do we get infected? How is it happening? So that you know how to be safe. Droplet infection, for example, is it enough to just turn away? Do you have to wear a face mask or just keep your mouth shut there and then? (No. 6, Female, 65 years old).

#### Understanding terms differently

Half of the participants did not use the terms “droplet transmission”, “airborne transmission” or “contact transmission”, which were used by the NIPH (Table 3).

**Table 3:** Descriptions derived from NIPH in January 2021

Terminology	Descriptions
Droplet transmission	People with COVID-19 emit droplets and particles from their noses and mouths that contain SARS-CoV-2
Contact transmission	Transmission occurs either directly through contact with a contagious person (for example, by a hug or handshake) or indirectly through contact with other surfaces contaminated with viruses (door handles, light switches, handrails)
Airborne transmission	Tiny virus-containing droplets/particles from the nose and mouth of an infectious person remain suspended in the air for a long time and move over longer distances

Instead, the participants emphasised behaviours for how transmission occurred, such as spitting, hugging, kissing, touching, or talking. The participants also often understood the terms differently from the formal NIPH definitions but did not have any awareness of doing so. Some of the participants associated droplet transmission with the transmission of saliva through kissing or touching, but they did not include droplets emitted from the infected person’s mucous membranes through coughing or sneezing. Some of the participants struggled to understand the difference between droplet and airborne transmission since both consist of droplets travelling through the air, leading to confusion when the media used these terms. Although the NIPH included hugging and kissing in its definition of (direct) contact transmission, these behaviours were not associated with “contact transmission” by any of the participants but rather with “droplet transmission”.

#### Acting on uncertain evidence

The participants had different perceptions of the uncertain evidence about virus transmission. For some of the elderly participants, the idea of airborne transmission and the possible severity of COVID-19 caused fear and isolation. For others, the uncertainty caused them to search for information online, which they used to make daily decisions.

One woman had worked in a surgical theatre and observed that people occasionally did not keep sufficient distance, did not wash their hands properly, and used face masks incorrectly. She had isolated herself at home for a year due to a fear of death and the dreaded health consequences of COVID-19 and to protect her elderly husband from infection:

Because it’s so cold here, I can see how big the breathing cloud is [outdoors]. It is far behind that person, and then I think if there are any droplets of virus in that cloud, one metre of distance is way too little… You cannot expect that everyone should work in a surgical theatre to understand this, but it’s not difficult to comprehend when you get it visualised like this (No. 6, Female, 65 years old).

Many of the participants expressed uncertainty over how long the coronavirus could survive on metal, clothes, and food. Most of the participants acted on generic advice from the health authorities, such as maintaining distancing, washing their hands, and avoiding touching public surfaces. However, they described a need for more specific knowledge to apply the advice to daily life. Some searched for information about virus transmission from nongovernmental and informal sources, despite its scientific uncertainty. One man avoided touching metal surfaces; one woman started disinfecting her mobile phone after watching a YouTube video that depicted contact transmission. A 79-year-old woman described washing her groceries to avoid transmission of the virus after reading a news article about the virus being able to survive on surfaces for three days:

I read online that it failed to stay alive for more than three days, but it could just be something people have written online, and I knew I could not trust it…I think it can be transmitted through food. I feel insecure when grocery shopping. For example, when I pick up clementines in the store, usually I just put them in a bowl in the living room, but now I wash the clementines before I put them in the living room because, I mean, there can be a little virus on them even if I have to peel them, and then it gets on my fingers. There are so many things I touch, and I think we are not careful enough when we buy food (No. 5, Female, 79 years old).

The participants expressed uncertainty over what happened in the “airspace” with respect to the time for which the virus survived in the air and the distance that it travelled.

### Risk mitigation

The second theme, *risk mitigation,* describes the public perceptions related to protecting oneself from being infected*.* Its three subthemes are affecting exposure to risk, learning about mitigation in different ways and being driven by symbolic values*.*

#### Affecting exposure to risk

All of the participants perceived that physical proximity to others increased the risk of exposure to the coronavirus and emphasised the NIPH’s advice about maintaining a 1-m distance. The participants believed that the risk for exposure could be affected by one’s culture (e.g., by participating in religious services or visiting pubs and cafés), hygiene knowledge or cognitive deficits (e.g., not understanding the severity of the risk, being a child or having dementia), attitudes (e.g., not caring about the risks to others and themselves), occupation (e.g., health care workers or bus drivers), activities (e.g., going to parties, consuming alcohol, riding on buses) and geographical factors (i.e., population density or cities with overcrowded housing). Environmental factors were also mentioned by some of the participants as vital to preventing the spread of the virus (i.e., indoors/outdoors, large rooms, ventilation, climate).

All of the participants stated that they reduced their exposure to risk by keeping their distance from other people and washing their hands with antibacterial gel. In contrast to the expert model, one participant claimed that the risk of being exposed to the coronavirus depended on static and categorical measures, such as talking to someone within a 1-m distance for more than 15 min; a 10-min conversation was considered safe.

However, generic advice to keep one meter distance was not sufficiently to make decisions in their everyday life. Some of the participants emphasised that receiving more information about risk activities and real-life information about infection locations, whether the activity was considered high risk, whether the outbreak was confined to a specific social environment, and whether it was under control could help them to make better decisions:

How much risk do I take if I go to the cinema? How risky is it to go ice skating on our lake? If I know that it is not wise to go ice skating there, then I might keep more distance. There are often such things someone does not quite know yet. More research is needed, or it [spread of infection] just has to happen, and then there will be results. When you do not know, it turns out that you just act and don’t think. One does not know what is worse than other things (No. 1, Female, 32 years old).

One 49-year-old man explained that receiving such information was important for him to make his own risk assessment and to be empowered to adapt:

This virus does not seem to disappear. Then, everyone needs to be able to assess the risk in the situation and make their own choices because that is what society is used to. It is life threatening, and we all make our own choices on where we take risks and where we do not take risks. That is the normal situation. This is not just a matter of sitting still in the boat until the storm is over. There are waves on the sea all the way, so we have to learn to navigate through the waves… I can make many safe choices without having to lock down my whole life and just sit inside a room if I had more knowledge about the kinds of situations in which infections occur (No. 4, Male, 49 years old).

#### Learning about mitigation in different ways

NIPH communicated *what* mitigation measures people should follow. The participants expressed the need to comprehend both the *what*, *why* and *how* of COVID-19 mitigation and expressed a variety of ways of learning about this topic.

Health risk information related to COVID-19 was sometimes perceived as confusing, complicated, rapidly changing, and variably practised across regions. Some participants expressed frustration with the political debate over pandemic management. Nevertheless, the participants acknowledged the complexity and uncertainty of pandemic management and expressed a generally high level of trust in the government and the health authorities’ (NIPH) risk communication since they were believed to provide the best foundation for decisions regarding COVID-19 mitigation.

Most of the participants reported that the government and the NIPH had been successful in communicating the core mitigation measures regarding *what* to do through mass media (e.g., press conferences, NIPH webpage) using familiar words and facts. However, many of the participants further noted the importance of comprehending the *why*, the reasons for the national and local restrictions and advice (e.g., why isolate, why vaccinate, why quarantine, why wear a face mask, why keep only one metre of distance and not two, why allow one activity and not another), as well as the effects of some of these measures, such as the effects of wearing a mask and getting the vaccine.

To accept action based on government advice, the participants needed to comprehend the reasons for the decisions, especially for restrictions and advice that changed over time:

I think it is very important that people understand why it is important to keep your distance, why it is important to wear a face mask because if you understand the reason why you should act the way you act, then it will be a little easier to keep your distance. I do it because the infection rates have to go down (No. 7, Female, 19 years old).

Many of the participants perceived that the government and the NIPH managed to explain the reasons for the advice and the restrictions, while others required deeper comprehension. Some of the participants sought informal sources, such as specific journalists, Snapchat, or YouTube videos, in which complex science information was translated into comprehensible, actionable and clear messages. Others sought primary sources and scientific evidence verified by multiple sources (e.g., scientific articles, WHO and NIPH webpages) because they endeavoured to understand the foundational evidence for the decisions made by the government and thereby to comprehend the reasons for the government’s restrictions and mitigation advice:

It is easier to rely on information that has been verified from several sources to understand the motivations of those who provide the information. I appreciate clear and in-depth information, and that it is really the web and things that are published from official sources, and of course, you have to take it for what it is. But then you have the opportunity to assess it. If it comes in a Snapchat message, it would not have helped me very much. I prefer real, verifiable information, and I would rather have it written than in videos (No. 4, Male, 49 years old).

Some of the participants felt overloaded with information related to restrictions, and they were not able to remain updated, focusing instead on information relevant to their daily lives or work situations, and they expressed a need for practical yet correct information about *how* to implement the mitigation measures (e.g., how to maintain distance on a bus, when to quarantine, how to interpret travel rules):

I would like information on how they think I should keep one metre away on a bus because when you sit right in front of someone, it is not one metre. You have to try around rush hour, but it’s a bit difficult. You structure the day about the same as others (No. 8, Female, 19 years old).

These participants insisted on a need for easily accessible, up-to-date online information available to comprehend the messages and on having someone to explain and interpret how to act on the restrictions.

#### Being driven by symbolic values

Simply understanding the importance of social distancing and hand washing was not always considered sufficient for the participants to protect themselves from the virus. Their daily decision making to reduce exposure to risk was sometimes driven by symbolic values rather than a lack of knowledge. The use of face masks and antibacterial gel in food stores were perceived as symbols of trust (i.e., ways to show others that they followed the mitigation rules), as well as symbols of distrust, since some people protected themselves due to a lack of trust in others complying with the infection control measures:

What to do, it’s a feeling you get. If you’re in a shop, you disinfect your fingers, and you may not have touched anything, and then you go into a new store, and you disinfect your fingers again. I feel that’s maybe done more for the public, those around me. But it is based on how I look at people who do not do the same because I do not know if they have been to any other places before. So you kind of feel that you are a bit obliged to do it, even if you have already done it. But it is to set an example and show that you do it yourself (No. 11, Male, 37 years old).

### Consequences of COVID-19

The third theme, *the consequences of COVID-19,* describes the public perceptions related to individual and societal consequences of the pandemic*,* divided into three subthemes: building situational awareness, perceiving personal health consequences differently and emphasising secondary consequences.

#### Building situational awareness

Some participants found that an early understanding that “this is not a seasonal flu” was important to understanding the severity of the risk.

First and foremost, it is important to really understand the seriousness, and that happened when we went into lockdown. Then, you understood quite quickly that this is not really the flu. It’s a little more serious (No. 8, Female, 19 years old).

All of the participants believed that COVID-19 had more serious health consequences than seasonal flu. With regard to contagion, only half of the participants believed the coronavirus was more contagious than the seasonal flu. Compared with the seasonal flu, COVID-19 was perceived to cause greater mortality and morbidity for those at high risk and even for healthy individuals. The variable courses of illness, from being asymptomatic to suffocation and death, even for healthy people, caused some of the participants to perceive the individual health risk as unpredictable and severe for all age groups. Lack of immunity, the potential for anyone to become dangerously ill, the lack of a vaccine and the global spread of the virus were reasons that COVID-19 was perceived as more severe than the seasonal flu.

The participants reported difficulties assessing the contagiousness of the coronavirus due to a lack of wider generic knowledge about viruses and their contagiousness. Furthermore, many described their perception of the contagiousness of COVID-19 changing during the pandemic. Furthermore, the emergence of viral variants demanded new understanding and revisiting of assumptions. The participants’ perceptions about contagiousness were affected and constantly changed by the information that they received from the government, health authorities and other sources of information.

The participants mentioned the consequences for individuals at risk for severe consequences due to catching COVID-19 and the consequences for society as vital to their situational awareness. Several of the participants perceived that the main danger of the virus was overload on the health care system and loss of control of the spread, and they described catastrophic scenarios from other countries:

If too many of us become ill, the more people will need intensive care and a respirator. I think the authorities are most afraid of the burden on the healthcare system. This is the major consequence that we see from other countries. I think the authorities are very concerned about keeping control so that we do not have a burden in society where ambulances have to stand in line to get patients in (No. 2, male, 67 years old).

Protecting older people and people with underlying diseases were the participants’ main motivations for undertaking infection mitigation measures. Having a family member who was at high risk for becoming severely ill or dying due to COVID-19 changed their views about the risk.

Understanding the individual and societal consequences of COVID-19 was important for building situational awareness of the severity of the crisis and creating motivations to respond, both in the response and in the long-term phase of the pandemic. However, many participants struggled to remain compliant after living almost a year under mitigation measures. They struggled to keep up with the constant changes in local and national restrictions, felt disengaged when watching the same types of press conferences with the same spokespersons talking about infection numbers in front of the same backgrounds and felt fatigued by listening to the same messages that described maintaining social distancing and using face masks. Many wanted the government to create awareness that the pandemic could last for a long time, and they required emphatic acknowledgement of citizens’ efforts so that they would be able to remain motivated until the population had been vaccinated. Some described a need for mental visualisation of an end to the crisis or believable messages about social distancing and lockdowns well into the future.

Half of the participants expressed that the basic reproduction number (*R*-value), which was repeatedly communicated in the media, helped them to build situational awareness about the risk of losing control of the outbreak; however, they complained of a lack of knowledge of the full meaning of the *R*-value. Some misunderstood the term as a correlation coefficient or the number of people reinfected with COVID-19.

The term “exponential growth” was perceived as a difficult concept to comprehend, and the participants stated that communications about the rapid spread of the virus and its consequences were more important than the use of the actual scientific term “exponential growth”.

#### Perceiving personal health consequences differently

The participants had a variety of perceptions of communications regarding individual health consequences and the disease itself. Some of the participants wanted to know more about the disease and its long-term health consequences; others reported that this type of information elicited fear and excessive worry.

A 34-year male participant claimed that knowledge about long-term consequences was important to perceive the risk seriously:

What I miss most is information about the long-term effects of getting COVID-19 because I know nothing about it. I know what I am supposed to do, or really, I know quite a lot about what I should do, but I don’t know anything about the long-term damage for the younger population. If we could also get long-term damage from it, then I think people would have taken it even more seriously (No. 14, Male, 34 years old).

The participants’ varying perceptions of personal health consequences were illustrated by two participants who described their reactions to the same documentary about elderly people hospitalised with COVID-19. Watching the documentary made one of the participants, a 65-year-old woman, so fearful that she had isolated herself in her home, afraid that the virus would kill the entire population of sick and elderly people. Another viewer, a 27-year-old man, claimed that the documentary had made him feel better informed about the possible consequences of the virus for the elderly members of his family:

I don’t want information about personal health consequences. I feel like I know it’s [COVID-19] very dangerous. I have seen the documentary *Helene Checks In* from a corona ward. After that, I was really scared. Since then, we have been almost completely isolated because it was absolutely cruel. It’s worse than COPD. They cannot breathe more than one way, and I saw the panic in their eyes when they tried to talk. It’s a terrible disease (No. 6, Female, 65 years old).

The *Helene Checks In* documentary made me think that it could have been very serious, especially if I was older, or for my parents because then I could see the outcome for older patients, especially an older man. He seemed very nice and upbeat, but he could not breathe and talk due to his respiratory problems. It made me think of my family then (No. 10, Male, 27 years old).

#### Emphasising secondary consequences

The participants noted the secondary consequences when discussing the major risk of COVID-19. They worried about personal and global economic consequences due to the loss of income and jobs and the consequences for the social lives and mental health of children, elderly people, and students:

What’s important is that the wheels keep going because being a farmer and a gardener, the biological processes go on continuously, whether its corona or not. The plants are growing, and the cows need to be milked (No. 13, Male, 51 years old).

Many struggled with loneliness, isolation, and a loss of freedom, and they were tired of living under strict infection control measures:

This is rough for single people, and they don’t have to be old. Many who have kids at home and try to manage this, they are having a hard time. And not to get out and touch a living human being is tough. I have some unmarried and widowed friends. They cannot even touch a man. That’s actually what life is about (No. 5, Female, 79 years old).

As the pandemic dragged on, some of the participants reported that they struggled with socially distancing from friends and from relatives. Some of the participants described making trade-offs between competing goals and values (i.e., reducing exposure to the virus and seeing friends and family). A woman stated that she regularly had her grandchildren over for visits, even if they had cold symptoms. She understood that she was exposing herself to risk, but their childhood years were so important to her:

If I see people coughing, I turn away. I stay away from people who have even small symptoms of disease, even though I have to admit that I have had my grandchildren over for visit with symptoms. I think it’s just a little cold. It is a qualified choice I make then. I understand that I am exposing myself to risk. When the minister of health said we should not be with grandchildren, I thought that I cannot stand a year without cuddling with my grandchildren (No. 2, Female, 67 years old)

### Comparation between the lay model and the expert model

The lay model (the public model) and the expert model (NIPH messages) displayed in Table 4 shows how experts and the public perceive information differently and identifies decision-relevant information missing in people’s mental models and topics that the participants themselves identifies. The messages were communicated by NIPH at the time of data collection (January-March 2021).

**Table 4:** Expert and lay model regarding COVID-19 health risk

NIPH messages	Public perceptions, actions and ways of learning
*Virus transmission modes* Relevant transmission modes: • droplets • air • contacts	Variably mentioned: • droplets • air • or/and contact Also mentioned: • food • clothes • faeces
*Dominant transmission route* • Droplet transmission most likely/significant • Airborne transmission and contact transmission exist but are nonsignificant	• Most did not separate between probable/less probable routes of transmission • Believed in multiple equal important ways of transmission
*Symptomatic, asymptomatic, presymptomatic spread* • Can be infected with and transmit SARS-CoV-2 virus without developing COVID-19 • Can be symptomatic carriers with COVID-19 • People with COVID-19 are most contagious for 1–2 days before the onset of symptoms and in the first days after the onset of symptoms	• Emphasise symptomatic transmission • Being sick without symptoms not mentioned by most but emphasised as vital to understand why you should keep distance and quarantine
*Terminology* • Terminology used on website: contact transmission, droplet transmission, airborne transmission	• Terms not used by most of the participants • Terms understood differently • Talked about transmission in relations to behaviours for how transmission occurred: • spitting • hugging • kissing • touching • talking
*Virus survival* • The virus can survive on surfaces from a few hours to several days • Depends on the amount of virus, temperature, sunlight, and humidity • The role that virus survival on surfaces plays in causing infection in humans is uncertain and constantly changing • Poorly ventilated rooms increase the concentration of particles containing the virus	• Acted on evidence from informal sources, e.g., three-day survival on surfaces • Few mentioned wind, climate and ventilation affected virus spread • Many wanted more knowledge about how far droplets spread and how long they could survive in the air
*Basic infection prevention measures* • Maintain social distancing, have fewer contacts, maintain hand hygiene and cough etiquette and use of face masks when not able to keep a distance	• All were informed about the main mitigation measures • Some people wanted to understand *why* certain behaviour and activities were considered high risk, others preferred simple, clear messages explaining *what* to do and *how* to protect themselves • Some sought informal sources to better comprehend the *why* • Easily accessible, up-to-date online information • Need someone to explain and interpret restrictions • Sometimes enacting mitigation measures was a symbolic action
*Safe distance* • The amount of virus exposed at distances of more 1–2 m would rarely be sufficient to cause infection • Mainly infected within 1–2 m reach from infected person • Keep one metre of distance • The greater that the distance that you keep is, the less that the probability is that you will be exposed to infection	• None of the participants talked in terms of probabilities • One of the participants misunderstood the 1-m rule as a clear boundary between safe and unsafe distances • Did not understand why 1 m and not 2 m
*Risky activities* • Risky activities due to increased expulsion of aerosols and/or being close to others are ◦ Pubs ◦ Travelling ◦ Exercise centres ◦ Poorly ventilated rooms	• Increased risk of being physically close to others was well understood • Struggling to understand why some activities were not allowed • Some wanted information about risky situations and risky localisations
*Protecting others* • The virus is possibly deadly for the oldest and some groups of people with chronic diseases	• All participants understood their collective responsibility to protect others
Contagiousness • The R-number is how many persons that one corona infected person infects further • A person infected with the coronavirus infects an average of 2–3 others, while one person with the flu infects 1–2 others	• COVID-19 contagiousness was underestimated by all of the participants • To comprehend the contagiousness of the virus, they had to understand that this disease was not influenza
*Control of the spread* • The *R*-value was communicated in terms of numbers, but exponential growth was not explained to the public	• The R-number was perceived as a good indicator regarding the control of the spread • The R-number was often misunderstood • No one understood exponential growth correctly
*Consequences of the pandemic* • NIPH communicated the health effects for the individual	• After one year with pandemic restrictions, most emphasised secondary consequences (e.g., economy, mental health) • Information about health consequences produced panic in some interviewees and awareness in others • There were daily trade-offs between social life and the risk of becoming ill

## Discussion

Our study found that communicating complex medical information about risk and uncertainty is complicated by people perceiving and acting on the same messages differently, having varying needs to learn about pandemic health risks, and having different preferences regarding information and modalities. Information about health consequences caused some people to panic and others to feel empowered. Terms related to virus transmission, exponential growth and the *R*-value were found to be difficult for many people to comprehend in the ways in which they were communicated. Some of the participants wanted simple rules to follow; others preferred to understand *why* they are important. Finally, we found that people’s mental models changed as they learned new information.

### Public mental model

On the whole, we found that participants reported high levels of trust in governmental and NIPH advice and reported being compliant. A high level of trust in authorities is associated with adherence to self-protective measures during pandemics [31]. Compared to other OECD countries, Norway is a high-trust society [32]; a recent study found that 96% of the participants reported trusting health authorities [33]. Trust in the health authorities was also high and stable during the first year of the COVID-19 pandemic; between 80 and 90% of the population expressed a high level of trust in the health authorities [34] and with the NIPH [35]. Despite high trust in formal sources and self-reported adherence, many of the participants searched for information from other sources since they lacked sufficient information to comprehend the risk. In particular, we identified knowledge gaps related to: 1) probability and modes of virus transmission; 2) the underlying principles explaining virus survival in the environment; 3) the probability of being infected decreasing with distance; 4) why and how to implement the rules; 5) the governmental strategies when implementing infection prevention at the group level; 6) why some activities are risky and current risky situations; and 7) contagiousness and the exponential growth of COVID-19.

New topics were identified by the public participants but not addressed by the NIPH, e.g., talking about transmission in terms of behaviours causing the transmission, symbolic values for mitigation behaviours, creating situational awareness by understanding that COVID is not influenza and making trade-offs between social life and the risk of being infected. These topics inform the development of theory regarding mental models and how people make decisions in pandemics.

The mental models approach to risk communication has been strongly inspired by cognitive psychology [3] and studies by Tversky and Kahneman [36]. According to Tversky and Kahneman [36], the way in which people think about a phenomenon tends to persist despite the availability of new information. People tend to filter new information according to its congruence with their existing understandings, beliefs and values; this process is conceptualised as *anchoring bias* [36]. Previous mental model studies of epidemic and pandemic risks have found that how people think about a health risk (i.e., Zika, COVID-19, pandemic influenza) is understood through their existing mental models and tends to persist, even when new information is available [15, 17]. Seasonal influenza has been most commonly used as a comparison with COVID-19 [37]. Nevertheless, our study found that, while people used the seasonal flu as a reference point to understand COVID-19 (i.e., anchoring bias), some people experienced seasonal flu as an inadequate foundation from which to comprehend COVID-19 risk. Despite having high trust in the Norwegian health authorities’ pandemic risk communication, they searched for other sources to comprehend the risk as fully as possible. They experienced that their perceptions regarding the risk related to the coronavirus were modified by changing information, indicating continual refinement of mental models and an *ongoing situational awareness* [38]. This dynamic approach to sensemaking could be vital for pandemic health risks since the available evidence and the expert model continue to change [1]. This study cannot specifically identify reasons why some people were less susceptible than others to anchoring bias. One explanation might be related to their motivations to seek information, associating people with a “high need for cognition” [39] with less susceptibility to framing effects [40]. Considering the dynamic characteristics of pandemic threats and infectious disease outbreaks [41], future mental model studies should describe how people adapt differently to new information, what they need to modify their preexisting beliefs, and how they are best supported, rather than how their decision-making fails to face this complexity.

This study suggests that some people made trade-offs related to quality of life rather than the risk of being infected (*longevity of life)* in the long term of the pandemic [42]. Being close to grandchildren, friends and family is important to quality of life, and people might prioritise these values above social distancing despite understanding the personal risk of being infected and the social risk of spreading the virus. Emotions play a role in decision-making regarding risk [43, 44], and our emotional responses are affected by our values [42]. Jones et al. [3] argued that peoples’ differing values must be considered in the elicitation of mental models, and our study supports that values and emotions are important to explore in descriptive decision theory approaches to risk communication since values and emotions affect the way in which people make decisions and how they perceive their world.

### Implications: supporting people in making informed decisions

The intent of the mental models approach is to identify the information that the public requires to make an informed decision [7]. By comparing the public mental models with the expert model, we identified the gaps that could be closed through communication interventions. Our study emphasises some considerations related to the use of technical language, empowering people, and creating awareness.

First, the findings indicate that people attach different meanings to the words used by Norwegian health experts in the NIPH. People understand virus transmission in terms of the behaviours causing the transmission of the virus and are unfamiliar with medical terms (droplets, airborne, or contact/surface transmission). The *R*-value and exponential growth are easily misunderstood. The media and health authorities might be unaware that they are using jargon, or they might overestimate people’s scientific literacy and health literacy; therefore, they might overestimate the ability of people to understand the terminology that is used [45]. Ironically, the use of both jargon and oversimplified wording could lead to miscommunications [46]. Communication could address this gap by educating people about what the *R*-value *is;* what factors affect these values; how contact, droplets and airborne transmission are different but interconnected; and which factors affect exposure to risk.

Second, the study findings indicate that, while people might be well informed about the required preventive measures of physical and social distancing, they might not necessarily comprehend why they have to keep their distance or how best to protect themselves. To make independent and informed decisions regarding exposure to risk, some people require more information about risk activities and risk locations and modes of virus transmission. Furthermore, people need information that mitigation rules is based on “probabilities”, e.g., protecting oneself from the most probable mode of virus transmission and that keeping two meters instead of one meter distance is about reducing the probability of being infected. Rather than *what* and *how*, they also need the *why*. Some people benefit from learning which certain involve a high risk and why and from receiving updated information about where the risk might be higher in the long-term phase of the pandemic. Such knowledge empowers people to make independent choices without harming others or imposing an undue collective burden [47]. However, empowerment depends on people’s capacity to act autonomously. Information that is difficult to comprehend can increase inequality for people who do not have access or the capacity to understand the information [48]. Thus, this study supports that there is no “one-size-fits-all” approach to public risk communication [49]. Communications must be targeted both to those who requires clear massages about *what* and *how* of communication and to those who also need the *why*.

Third, the study suggests that being informed in a way that facilitates comprehension of what is happening and how to avoid dangerous situations is important for initiating the response to risk and sustaining motivation. According to Endsley [38], these ideas are related to *situational awareness*, which is ongoing awareness of one’s environment and especially of events that one must understand. High levels of situational awareness have been associated with social distancing [35, 50] and hand-washing behaviour [51]. However, efficient and ethically sound health risk communication requires a balance between providing people with information that strengthens their awareness without sowing panic [52]. Although *functional fear* of COVID-19 has been found to encourage public health-promoting behaviours [53], an excessive fear of COVID-19 could increase maladaptive behaviours in the long term [54]. This study finds that information about health consequences caused panic for some and awareness for others, emphasising the complexity of communicating health consequences to the public in the hope of creating awareness [52]. Policy makers and communication creators must be aware that information about future scenarios leads to a variety of understandings and reactions.

### Limitations

The use of video can affect the establishment of a relationship with those who are uncomfortable using video conferences [55]. To limit this risk and increase trust and candour, we asked the participants to choose their preferred mode of communication (telephone or video) [19]. To facilitate quick sampling, we used extended networks and nongovernmental organisations. The recruitment followed a purposive sampling strategy to recruit participants with a diversity of experiences (i.e., men and women, various ages and levels of education, different geographical regions in Norway and diverse occupational areas). This approach provided us with a more specific sample than we would have obtained with a convenience sample, which, according to Malterud [56], enhances the information power (the information that the sample holds). The total sample was considered sufficient *to explore* a variety of mental models related to COVID-19 risk and mitigation in the population and to develop a rich understanding of the topic [56]. To describe mental models for specific populations in various contexts, more research is needed. Finally, this study employed a qualitative approach to understand and compare the mental models of the public and experts with respect to perceiving and acting on health information related to COVID-19. Understanding the prevalence of these mental models within the wider population would require larger survey studies and experimental research to test communication interventions [7].

## Conclusion

Our findings suggest that the public varies in its knowledge of how COVID-19 is transmitted and why some activities are riskier than others. People comprehend and act on complex information differently and assign different meanings to the jargon used in risk communication. While some people called for simple behavioural rules to follow, others report that not comprehending *why* limited their independent decision-making in contexts in which following rules (e.g., maintaining social distance) was inadequate. To empower people to make informed and independent decisions, risk communication could benefit from targeted interventions that translate scientific information related to modes of virus transmission and activities with increased exposure to risk. Targeted communication requires better consideration of the media channels and the audio-visual languages best suited to the intended audience. Furthermore, when a new virus strikes, people need information that enables them to modify their mental models regarding why they need to act differently. In the COVID-19 pandemic, the comprehension of the societal consequences related to the contagious nature of the virus and the protection of individuals at high risk are of great importance.

## Supplementary Information

**Additional file1.** Semistructured interview guide.

## Data Availability

Anonymised data used and/or analysed during the current study are available from the corresponding author on reasonable request.

## References

[1] Koffman J, Gross J, Etkind SN, Selman L (2020). Uncertainty and COVID-19: how are we to respond?. J R Soc Med.

[2] Li Q, Guan X, Wu P, Wang X, Zhou L, Tong Y (2020). Early Transmission Dynamics in Wuhan, China, of Novel Coronavirus-Infected Pneumonia. N Engl J Med.

[3] Jones NA, Ross H, Lynam T, Perez P, Leitch A. Mental models: an interdisciplinary synthesis of theory and methods. Ecol Soc. 2011;16(1):46.

[4] Johnson-Laird PN. Mental models: Towards a cognitive science of language, inference, and consciousness. Cambridge/Massachusetts: Harvard University Press; 1983.

[5] Elsawah S, Guillaume JH, Filatova T, Rook J, Jakeman AJ (2015). A methodology for eliciting, representing, and analysing stakeholder knowledge for decision making on complex socio-ecological systems: from cognitive maps to agent-based models. J Environ Manage.

[6] Breakwell GM (2001). Mental models and social representations of hazards: the significance of identity processes. J Risk Res.

[7] Morgan MG, Fischhoff B, Bostrom A, Atman CJ. Risk communication: A mental models approach. Cambridge: Cambridge University Press; 2002.

[8] Berg SH, O’Hara JK, Shortt MT, Thune H, Brønnick KK, Lungu DA (2021). Health authorities’ health risk communication with the public during pandemics: a rapid scoping review. BMC Public Health.

[9] Boase N, White M, Gaze W, Redshaw C (2017). Evaluating the Mental Models Approach to Developing a Risk Communication: A Scoping Review of the Evidence. Risk Anal.

[10] Galada HC, Gurian PL, Corella-Barud V, Pérez FG, Velázquez-Angulo G, Flores S (2009). Applying the mental models framework to carbon monoxide risk in northern Mexico. Rev Panam Salud Publica.

[11] Downs JS, Murray PJ, de Bruin WB, Penrose J, Palmgren C, Fischhoff B (2004). Interactive video behavioral intervention to reduce adolescent females’ STD risk: A randomized controlled trial. Soc Sci Med.

[12] Newby KV, French DP, Brown KE, Wallace LM (2013). Beliefs underlying chlamydia risk appraisals: the relationship with young adults’ intentions to use condoms. J Risk Res.

[13] Chakrapani V, Newman PA, Singhal N, Nelson R, Shunmugam M (2013). “If It’s Not Working, Why Would They Be Testing It?”: mental models of HIV vaccine trials and preventive misconception among men who have sex with men in India. BMC Public Health.

[14] De Vries M, Claassen L, Te Wierik MJ, Coban F, Wong A, Timmermans DR (2020). Meningococcal W135 disease vaccination intent, the Netherlands, 2018–2019. Emerg Infect Dis.

[15] Southwell BG, Kelly BJ, Bann CM, Squiers LB, Ray SE, McCormack LA (2020). Mental Models of Infectious Diseases and Public Understanding of COVID-19 Prevention. Health Commun.

[16] Downs JS, de Bruin WB, Fischhoff B (2008). Parents’ vaccination comprehension and decisions. Vaccine.

[17] Yang H, Pang X, Zheng B, Wang L, Wang Y, Du S (2020). A Strategy Study on Risk Communication of Pandemic Influenza: A Mental Model Study of College Students in Beijing. Risk Manag Healthc Policy.

[18] Balog-Way D, McComas K, Besley J (2020). The Evolving Field of Risk Communication. Risk Anal.

[19] de Bruin WB, Bostrom A (2013). Assessing what to address in science communication. Proc Natl Acad Sci.

[20] Røislien J, O'Hara JK, Smeets I, Brønnick K, Berg SH, Shortt MT (2022). Creating Effective, Evidence-Based Video Communication of Public Health Science (COVCOM Study): Protocol for a Sequential Mixed Methods Effect Study. JMIR Research Protocols.

[21] Berg SH, Shortt MT, Røislien J, Lungu DA, Thune H, Wiig S. Key topics in pandemic health risk communication: A qualitative study of expert opinions and knowledge. medRxiv. 2022. 10.1101/2022.03.28.22273033. Accessed 1June 2022. 10.1371/journal.pone.0275316 PMC9524709 36178941

[22] NIPH. Nordmann bosatt i Italia smittet av koronavirus National Institute of Public Health 2020 [Available from: https://www.fhi.no/nyheter/2020/nordmann-bosatt-i-italia-smittet-av-koronavirus/.

[23] Government.no. The Government is establishing clear quarantine and isolation rules 2020 [Available from: https://www.regjeringen.no/en/aktuelt/the-government-is-establishing-clear-quarantine-and-isolation-rules/id2693647/.

[24] Ministry of Health and Care Services. Emergency preparedness plan for infection mitigation during the COVID-19 pandemic Regjeringen.no2020 [Available from: https://www.regjeringen.no/contentassets/73a60433276240bb9247a00ecc8b23c7/beredskapsplan-covid-19-oppdatert-desember-2020-hbrj.pdf.

[25] Worldometers. Reported Cases and Deaths by Country or Territory 2021 [Available from: https://www.worldometers.info/coronavirus/#countries.

[26] Bruine de Bruin W, Fischhoff B, Brilliant L, Caruso D (2006). Expert judgments of pandemic influenza risks. Global Public Health.

[27] Fischhoff B, Downs JS, de Bruin WB (1998). Adolescent vulnerability: A framework for behavioral interventions. Appl Prev Psychol.

[28] NIPH. Coronavirus – facts, advice and measures Norwegian Institute of Public Health 2020 [Available from: https://www.fhi.no/en/op/novel-coronavirus-facts-advice/.

[29] Government.no. National measures that apply to everyone from 5th November Government.no2020 [Available from: https://www.regjeringen.no/en/topics/koronavirus-covid-19/national-measures-that-apply-to-everyone-from-5th-november/id2783881/.

[30] NIPH. Facts about the SARS-CoV-2 virus and COVID-19 disease Norwegian Institute of Public HEalth 2020 [Available from: https://www.fhi.no/en/op/novel-coronavirus-facts-advice/facts-and-knowledge-about-covid-19/facts-about-novel-coronavirus/?term=&h=1.

[31] Bish A, Michie S (2010). Demographic and attitudinal determinants of protective behaviours during a pandemic: A review. Br J Health Psychol.

[32] OECD.org. Trust in Government OECD2021 [Available from: http://www.oecd.org/gov/trust-in-government.htm.

[33] Sætrevik B (2021). Realistic expectations and pro-social behavioural intentions to the early phase of the COVID-19 pandemic in the Norwegian population. Collabra Psychology.

[34] Coronavirus Commission. NOU 2021-The Norwegian Government's Management of the Coronavirus Pandemic. [Cited 2021 May 1]. 2021. https://www.regjeringen.no/no/dokumenter/nou-2021-6/id2844388/. Accessed 1 May 2021.

[35] Zickfeld JH, Schubert TW, Herting AK, Grahe J, Faasse K (2020). Correlates of health-protective behavior during the initial days of the COVID-19 outbreak in Norway. Front Psychol.

[36] Tversky A, Kahneman D (1974). Judgment under uncertainty: Heuristics and biases. science.

[37] de Bruin WB, Carman KG, Parker AM (2021). Mental associations with COVID-19 and how they relate with self-reported protective behaviors: A national survey in the United States. Soc Sci Med.

[38] Endsley M.R. Toward a Theory of Situation Awareness in Dynamic Systems. Human Factors. 1995;37(1):32–64.

[39] Cacioppo JT, Petty RE, Feng KC (1984). The efficient assessment of need for cognition. J Pers Assess.

[40] Carnevale JJ, Inbar Y, Lerner JS (2011). Individual differences in need for cognition and decision-making competence among leaders. Personality Individ Differ.

[41] Salajan A, Tsolova S, Ciotti M, Suk JE (2020). To what extent does evidence support decision making during infectious disease outbreaks? A scoping literature review. Evid Policy.

[42] Berry D. Risk, communication and health psychology. UK: McGraw-Hill Education; 2004.

[43] Loewenstein GF, Weber EU, Hsee CK, Welch N (2001). Risk as feelings. Psychol Bull.

[44] Hastie R (2001). Problems for judgment and decision making. Annu Rev Psychol.

[45] Castro CM, Wilson C, Wang F, Schillinger D (2007). Babel babble: physicians' use of unclarified medical jargon with patients. Am J Health Behav.

[46] Nerlich B, Koteyko N, Brown B (2010). Theory and language of climate change communication. Wiley Interdisciplinary Rev.

[47] Oxman AD, Fretheim A, Lewin S, Flottorp S, Glenton C, Helleve A (2022). Health communication in and out of public health emergencies: to persuade or to inform?. Health Res Policy Syst.

[48] Tengland P-A (2012). Behavior Change or Empowerment: On the Ethics of Health-Promotion Strategies. Public Health Ethics.

[49] World Health Organization (2017). Communicating risk in public health emergencies: a WHO guideline for emergency risk communication (ERC) policy and practice.

[50] Qazi A, Qazi J, Naseer K, Zeeshan M, Hardaker G, Maitama JZ (2020). Analyzing situational awareness through public opinion to predict adoption of social distancing amid pandemic COVID-19. J Med Virol.

[51] Liao Q, Cowling B, Lam WT, Ng MW, Fielding R (2010). Situational Awareness and Health Protective Responses to Pandemic Influenza A (H1N1) in Hong Kong: A Cross-Sectional Study. PLoS ONE.

[52] Guttman N, Lev E (2021). Ethical Issues in COVID-19 Communication to Mitigate the Pandemic: Dilemmas and Practical Implications. Health Commun.

[53] Harper CA, Satchell LP, Fido D, Latzman RD (2020). Functional fear predicts public health compliance in the COVID-19 pandemic. Int J Ment Heal Addict.

[54] Garfin DR, Silver RC, Holman EA (2020). The novel coronavirus (COVID-2019) outbreak: Amplification of public health consequences by media exposure. Health Psychol.

[55] Melis G, Sala E, Zaccaria D. Remote recruiting and video-interviewing older people: a research note on a qualitative case study carried out in the first Covid-19 Red Zone in Europe. Int J Soc Res Methodol. 2022;25(4):477–82.

[56] Malterud K, Siersma VD, Guassora AD (2016). Sample Size in Qualitative Interview Studies: Guided by Information Power. Qual Health Res.

