# Supplementary material for: Differences in comprehending and acting on pandemic health risk information: a qualitative study using mental models
Source: BMC Public Health. 2022 Jul 29;22:1440. doi: 10.1186/s12889-022-13853-y (PMC9334540; doi:10.1186/s12889-022-13853-y)
Supplement: Supplementary file 1 — Additional file1. Semistructured interview guide. [file 12889_2022_13853_MOESM1_ESM.pdf]

Additional file 1 Semistructured interview guide

Information

This interview is part of our research at the University of Stavanger on risk communication about the coronavirus SARS-CoV-2, which causes the COVID-19 disease. You should not provide any information about your own illness or state of health in this interview. I am going to use a tape recorder, and the audio file from this interview will be stored in an encrypted area on a PC that is protected by a password. Your name will be anonymised when the study is published. What you say will have no consequences for you, and no one other than the researchers involved in the project will have access to the raw data collected.

Demographics

First, I have some questions for you. These questions will be used to describe the sample of participants in this study.

- Where do you live?
- What is your age?
- What are your education and current occupation status?

Introduction

In this interview, I am interested in obtaining insight into how you think about prevention and risk for COVID-19. There are no right or wrong answers. I want you to tell me everything you think about regarding this topic.

- Can you tell me about the coronavirus and what risk it causes?
  - ➢ Is corona dangerous, and if so, how?
  - ➢ Can you tell me more about this danger?
  - ➢ What is important for you regarding COVID-19 risk?

Beliefs regarding the coronavirus and COVID-19

Virus transmission

- Can you tell me (more) about how people are infected by the coronavirus?
  - ➢ Means of transmission: droplet transmission, airborne transmission, contact transmission
- Can you explain how the virus spreads through XX?
- Have you heard about other ways in which the coronavirus spreads?
- Do you have any opinion regarding how sure or uncertain the government is regarding how the virus spreads?
  - ➢ What do you experience the government being sure/unsure about?

Exposure

- Is there anything affecting whether someone is more likely to become infected than others?
  - ➢ Is there anything that characterises these individuals?
  - ➢ Is there anything related to different conditions?
- How contagious is the coronavirus?

Consequences and health effects

- Do you require information about the consequences of the disease COVID-19?
  - What topics do you need more information about (regarding the consequences of COVID-19)?
- What have you heard could be the health consequences of contracting COVID-19?
  - You say COVID-19 can cause XX; can you tell me more about that?
- Do you have any opinions regarding how sure or uncertain the government is regarding the consequences of being infected by the coronavirus?
  - What do you experience the government being sure/unsure about?

Risk comparison

- Is COVID-19 an important risk in Norwegian society, or is it not very important?
  - Why is it an important risk?
- Can you give me an idea about what the health risk for COVID-19 is compared to other contagious diseases, such as the seasonal flu?
- How contagious is the coronavirus, compared to other infectious diseases, such as the seasonal flu?

Experiences and needs related to risk communication

Learning and prevention

- What have you heard that you can do to avoid being infected and avoid infecting others?
- What information do you need regarding virus transmission and mitigation?
  - Is there anything you would like to have more information about?

- How have you learned and heard about the coronavirus?
  - Where do you obtain information regarding the coronavirus?
  - What type of information do you trust regarding the coronavirus?
  - Are there any ways of communicating information regarding the coronavirus that are more or less beneficial for you?

- Have you watched any videos about the coronavirus and COVID-19?
  - Where have you seen these videos?
  - How did these videos affect you?

Formal risk communication

- What information have you received from the government regarding pandemic risk that you deemed important?
  - Is there any type of information regarding the coronavirus and COVID-19 that you believe has been difficult to comprehend?
  - How do you prefer to receive information regarding the coronavirus?

Jargon

I have some terms that I wonder if you have heard about or read. No worries if you have not heard about the topics; this is also useful for us to know. Can you tell me what you think about when you hear these concepts?

- Have you heard about exponential growth in relation to COVID-19?

    - Can you explain what exponential growth is?
    - Do you experience this concept as useful? Why or why not?
- Have you heard about the R value, or the reproduction rate, in relation to COVID-19?
    - Can you explain what the R value is?
    - Do you experience this concept as useful? Why or why not?

Video intervention

- At the end of this interview, I wonder whether you have any good advice for our research. project. We are developing a video to communicate pandemic risks to society. In your opinion, what should be the topic of this video?
